# Image-guided high-dose-rate brachytherapy for rectal cancer: technical note and first clinical experience on an organ-preserving approach

**DOI:** 10.1007/s00066-022-01931-4

**Published:** 2022-04-20

**Authors:** Maximilian Fleischmann, Markus Diefenhardt, Martin Trommel, Christian Scherf, Ulla Ramm, Georgios Chatzikonstantinou, Emmanouil Fokas, Claus Rödel, Nikolaos Tselis

**Affiliations:** 1 Department of Radiation Oncology, University Hospital Johann Wolfgang Goethe University Frankfurt, Theodor-Stern-Kai 7, 60590 Frankfurt am Main, Germany; 2 German Cancer Research Center (DKFZ), Heidelberg, Germany; 3 Partner Site Frankfurt am Main, German Cancer Consortium (DKTK), Frankfurt, Germany; 4 Frankfurt Cancer Institute, Frankfurt, Germany

**Keywords:** Rectal cancer, Radiotherapy, Brachytherapy, Endoluminal, Endorectal, Nonoperative management, Organ preservation, Complete response

## Abstract

**Purpose:**

As the population ages, the incidence of rectal cancer among elderly patients is rising. Due to the risk of perioperative morbidity and mortality, alternative nonoperative treatment options have been explored in elderly and frail patients who are clinically inoperable or refuse surgery.

**Methods:**

Here we present technical considerations and first clinical experience after treating a cohort of six rectal cancer patients (T1‑3, N0‑1, M0; UICC stage I-IIIB) with definitive external-beam radiation therapy (EBRT) followed by image-guided, endorectal high-dose-rate brachytherapy (HDR-BT). Patients were treated with 10–13 × 3 Gy EBRT followed by HDR-BT delivering 12–18 Gy in two or three fractions. Tumor response was evaluated using endoscopy and magnetic resonance imaging of the pelvis.

**Results:**

Median age was 84 years. All patients completed EBRT and HDR-BT without any high-grade toxicity (> grade 2). One patient experienced rectal bleeding (grade 2) after 10 weeks. Four patients (67%) demonstrated clinical complete response (cCR) or near cCR, there was one partial response, and one residual tumor and hepatic metastasis 8 weeks after HDR-BT. The median follow-up time for all six patients is 42 weeks (range 8–60 weeks). Sustained cCR without evidence of local regrowth has been achieved in all four patients with initial (n)cCR to date.

**Conclusion:**

Primary EBRT combined with HDR-BT is feasible and well tolerated with promising response rates in elderly and frail rectal cancer patients. The concept could be an integral part of a highly individualized and selective nonoperative treatment offered to patients who are not suitable for or refuse surgery.

## Introduction

Multimodal treatment strategies have enabled selective organ preservation and resulted in a paradigm shift in the management of rectal cancer. In patients with locally advanced rectal cancer, total neoadjuvant therapy (TNT) has significantly improved pathological complete response (pCR) and disease-free survival (DFS) rates, as recently demonstrated by the phase III randomized RAPIDO and PRODIGE-23 trials [1–3]. Given the morbidity of radical surgery, such as temporary/permanent colostomy, stool incontinence, and urinary and sexual dysfunction, a selective nonoperative management (NOM) approach offers an opportunity to avoid a negative and profound long-term impact on quality of life in patients with clinical complete response [4–6]. However, TNT concepts currently being investigated to improve functional outcomes and quality of life incorporate intensified chemotherapy regimens and are often not feasible in elderly and frail patients due to multiple comorbidities. Moreover, major surgery poses a high risk of perioperative complications and mortality in these patients [7–10].

Radiation therapy (RT) dose escalation is associated with increased tumor regression and improved response rates in rectal cancer. However, dose response analyses indicate that a biologically equivalent dose (EQD2) of 92 Gy is required to achieve pCR in approximately 50% of patients with locally advanced disease by RT alone [11, 12]. Emerging NOM and/or local excision (LE) approaches after RT alone have been reported for localized and early-stage disease [13, 14]. In this context, contact x‑ray brachytherapy (CXB), usually performed with 50-kV x‑rays, has shown local control rates up to 86% for selected T2‑3 tumors less than 3 cm in diameter [15–19]. Alternatively, endorectal high-dose-rate brachytherapy (HDR-BT) delivers a highly conformal dose distribution with steep dose gradients, also covering higher volumes and locally advanced tumors. Previous data on definitive RT consisting of external-beam radiation therapy (EBRT) followed by an endorectal HDR-BT boost have demonstrated promising local control rates and tolerable toxicity rates [20–25]. We here report our first experience on definitive RT combining EBRT and image-guided endorectal HDR-BT in a cohort of six elderly and frail rectal cancer patients not suitable for or refusing radical surgery.

## Materials and methods

### Treatment

The treatment regimen consisted of EBRT followed by a restaging assessment (RA) and sequential image-guided endorectal HDR-BT. EBRT was applied as intensity-modulated radiotherapy (IMRT) or volumetric modulated arc therapy (VMAT) and, if feasible, in prone position using a belly board. Patients received 30 to 39 Gy in 10 or 13 daily fractions, respectively. Initially, a dose of 30 Gy in 10 fractions was prescribed to evaluate tolerability in this vulnerable patient cohort. After 10 × 3 Gy proved to be not associated with increased toxicity and did not affect the feasibility of this combined approach (EBRT + HDR-BT), we increased the EBRT dose to 13 × 3 Gy. Clinical target volumes (CTV) included the primary tumor with margins, the involved lymph nodes, as well as the mesorectum, presacral, and internal iliac lymph nodes up to S2‑3 in low rectal tumors without suspected lymph node involvement, or the interspace, or L5-S1, respectively. The inferior border was at least 3 cm below the primary tumor. Bladder, small intestines, and the femoral head were defined as organs at risk (OAR).

Six weeks after EBRT and prior to the first HDR-BT, MRI of the pelvis and endoscopy were performed to evaluate treatment response and the residual extent of disease for target outlining. Patients were prepared for treatment with a whole-bowel irrigation to ensure clean intestines. During endoscopy, radiopaque CT markers were placed at the lateral, proximal, and distal margin of the residual tumor for target volume delineation and image guidance during treatment. A cylindrical intracavitary mold applicator (Elekta AB, Sweden) with eight radially shaped treatment catheters of 270 mm in length and 20 mm in diameter covered with an inflatable, semicircular balloon was used to perform HDR-BT. Patients were positioned on a dedicated brachytherapy table (Brachy T‑table, GfM mbH, Groß-Gerau, Germany), enabling force-free transfer and ensuring the same patient position and implant geometry during CT imaging and HDR-BT. After insertion of the applicator in left lateral position, the balloon was inflated with 20–30 ml water and iodine containing radiocontrast to guarantee a proper contact area and secure positioning in the rectum. The semicircular balloons further increase and visualize the distance between the target volume and the contralateral rectal wall. Next, a CT scan (1.5-mm slice thickness) was done in supine position to verify correct positioning and to perform three-dimensional (3D) treatment planning. The planning target volume (PTV) encompassed the residual gross tumor volume (GTV; equal to clinical target volume [CTV]) with margins of 5 mm in cranial, caudal, and lateral directions (PTV = GTV + 5 mm). The GTV was defined by radiopaque markers placed at the borders of the macroscopically visible tumor during endoscopy 6 weeks after EBRT. Final approval followed plausibility verification under consideration of pre-interventional MRI and endoscopy findings. Organs at risk included the bladder and rectum outside the PTV. Depending on the initial T category, dose was delivered at a depth of 5 mm (T1) or 10 mm (≥ T2) from the applicator surface.

**Fig. 1:**
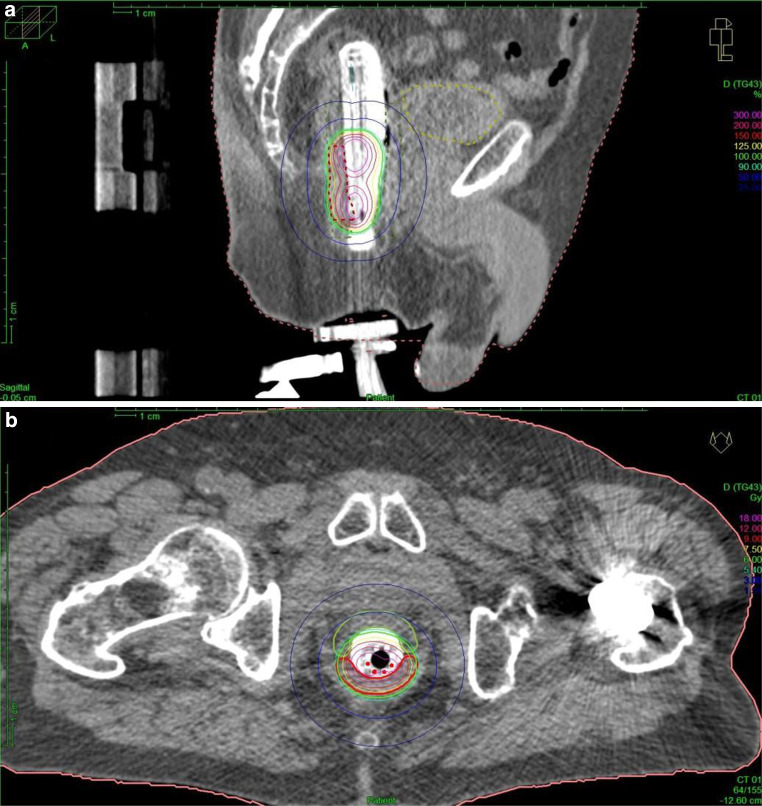
CT-based treatment planning of patient 6. Sagittal (**a**) and axial (**b**) images of CT-based treatment planning and distribution. A semicircular balloon filled with radiocontrast serves as a spacer to increase the distance between target volume and the contralateral rectal wall and ensures stable positioning and proper contact area. The applicator is additionally fixed to the treatment table by a specialized clamp. Isodose color code convention: *green* 6 Gy (100%), *red* 9 Gy (150%), *magenta* 18 Gy (300%, mucosa contact dose). Planning target volume (PTV) is also marked in *red*

Three-dimensional dose optimization was performed with Oncentra® Brachy (Elekta AB, Sweden). Fig. 1 represents an example dose distribution after CT-based treatment planning. The treatment plan was checked and adjusted prior to every fraction on a slice-by-slice basis. The prescribed HDR-BT dose of 6 Gy per fraction was delivered by a remote afterloading system (Flexitron, Elekta AB, Sweden) with an iridium-192 source and apparent initial source activity of approximately 370 GBq. Three fractions of HDR-BT were performed once weekly. None of the patients received concomitant chemotherapy.

## Results

### Patient characteristics

Six patients with histologically confirmed adenocarcinoma of the rectum (T1‑3, N0‑1, M0; UICC I-IIIB) were treated with EBRT followed by image-guided endorectal HDR-BT between August 2020 and September 2021. Median age was 84 years. All patients were assigned to primary NOM due to their comorbidities or refusal of surgery. The most common comorbidities leading to functional inoperability were chronic heart failure and severe chronic obstructive pulmonary disease (COPD). Staging was performed with endoscopy, magnetic resonance imaging (MRI) of the pelvis, and computed tomography (CT) scans of chest/abdomen. Endorectal ultrasound was used to differentiate between uT1 and uT2 tumors. Detailed patient characteristics are shown in Table 1**.**

**Table 1:** Patient characteristics

Patient	Sex	Age	TNM stage	mrMRF+	Distance from anal verge	ECOG performance status (Karnofsky)
1	Female	83	T1 N0 M0	–	2–3 cm	1(70)
2	Male	88	T2 N0 M0	No	5 cm	2 (60)
3	Male	80	T1 N0 M0	–	5 cm	1 (80)
4	Male	86	T3 N1 M0	Yes	3 cm	2 (60)
5	Female	75	T2 N0 M0	No	11–13 cm	2 (50)
6	Male	86	T2 N1 M0	No	< 1 cm	0 (90)

*mrMRF* mesorectal fascia status on MRI, *ECOG* Eastern Cooperative Oncology Group

### Dosimetric results

In total, EBRT (30–39 Gy in 10 or 13 fractions, respectively) and sequential HDR-BT with 6 Gy per fraction resulted in total in a median cumulative EQD2_α/β10_ of 61 Gy (range 48.5–66.3 Gy) at 5 mm or 10 mm depth, a median mean EQD2_α/β10_ of the PTV (m∑EQD2_α/β10_) of 76.5 Gy (range 68.6–88.3 Gy), and a median EQD2_α/β10_ at the mucosal surface level (c∑EQD2_α/β10_) of 146 Gy (range 95–168.3 Gy). Median PTV was 8.425 ccm (range 3.33–20.76 ccm). Table 2 summarizes the treatment parameters of all patients.

**Table 2:** Treatment characteristics

Patient	TNM stage	EBRT	HDR-BT	PTV depth	∑EQD2_α/β10_	m∑EQD2 _α/β10_	c∑EQD2 _α/β10_	PTV (ccm)
1	T1 N0 M0	30 Gy (10 × 3)	2 × 6 Gy	5 mm	48.5 Gy	68.6 Gy	95 Gy	3.33
2	T2 N0 M0	30 Gy (10 × 3)	3 × 6 Gy	10 mm	56.5 Gy	76.9 Gy	158.8 Gy	10.72
3	T1 N0 M0	39 Gy (13 × 3)	3 × 6 Gy	5 mm	66.3 Gy	76.5 Gy	136 Gy	4.19
4	T3 N1 M0	30 Gy (10 × 3)	3 × 6 Gy	10 mm	56.5 Gy	69.7 Gy	126.3 Gy	20.76
5	T2 N0 M0	39 Gy (13 × 3)	3 × 6 Gy	10 mm	66.3 Gy	88.3 Gy	168.3 Gy	6.13
6	T2 N1 M0	39 Gy (13 × 3)	3 × 6 Gy	10 mm	66.3 Gy	84.5 Gy	157 Gy	14.79

Average planning target volume (*PTV*) of all HDR-BT fractions

∑EQD2_α/β10_ = EQD2_α/β10_ EBRT + EQD2_α/β10_ HDR-BT at 5 mm or 10 mm m∑EQD2_α/β10_ = EQD2_α/β10_ EBRT + EQD2_α/β10_ mean dose HDR-BT c∑EQD2_α/β10_ = EQD2_α/β10_ EBRT + EQD2_α/β10_ HDR-BT contact dose/surface of rectal mucosa *EBRT* external beam radiation therapy, *HDR-BT* high-dose rate brachytherapy, *PTV* planning target volume

### Treatment compliance and toxicity

All patients received EBRT as planned without dose reduction or RT interruption. Toxicity of EBRT and HDR-BT was evaluated according to the Common Terminology Criteria for Adverse Events (CTCAE) version 5.0. Treatment was tolerated without any high-grade acute toxicity (CTCAE grade > 2) or other complications. Mild to moderate proctitis was the most frequently reported acute toxicity after EBRT, requiring symptomatic treatment only. Urinary toxicities were negligible. Apart from patient 1, all patients received three HDR-BT fractions. Placing the applicator was well tolerated with only minor discomfort and did not require anesthesia. After HDR-BT, patients reported minor rectal discomfort and minimally increased frequency of stools (< 4) per day. One patient had rectal bleeding (CTCAE grade 2) 10 weeks after treatment, requiring argon plasma coagulation. Table 3 provides an overview of acute and long-term toxicities.

**Table 3:** Acute and long-term toxicity after EBRT and HDR-BT

Patient	TNM	Acute toxicity (EBRT)	Acute toxicity (HDR-BT)	Long-term toxicity
1	T1 N0 M0	0	0	Diarrhea grade I
2	T2 N0 M0	0	1	No
3	T1 N0 M0	2	0	Diarrhea grade I Rectal hemorrhage grade I
4	T3 N1 M0	1	0	No
5	T2 N0 M0	2	1	No
6	T2 N1 M0	1	1	Fecal incontinence grade I Proctitis grade I

Acute toxicity refers to proctitis *EBRT* external beam radiation therapy, *HDR-BT* high-dose rate brachytherapy

### Clinical outcomes and follow-up

**Fig. 2:**
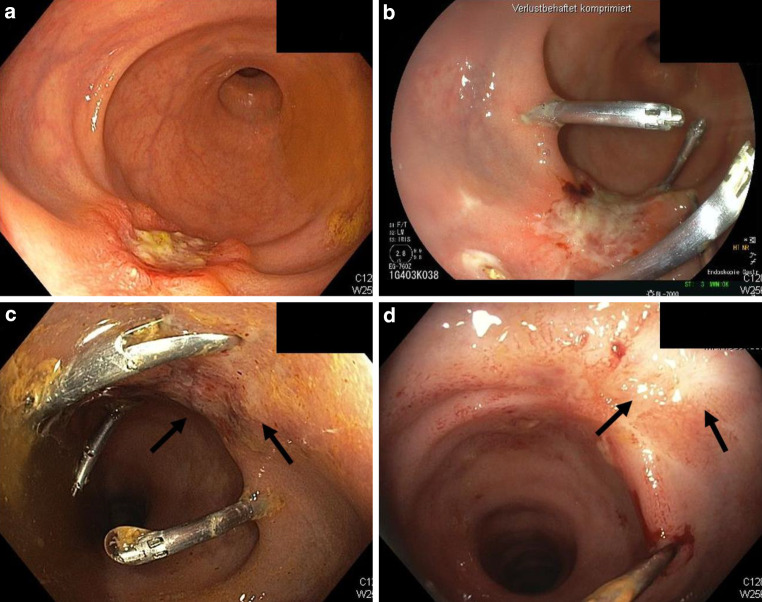
Endoscopic findings of patient 2. **a** Eight weeks after 10 × 3 Gy EBRT (external beam radiation therapy), a macroscopic tumor residue with a fibrinous layer appears in the distal rectum. Radiopaque CT (computed tomography) markers are placed to define the extent of the residual tumor in distal, proximal, and lateral directions. **b** After two weekly applied fractions of HDR-BT (high-dose rate brachytherapy) of 6 Gy each, a first endoscopy shows a clearly regressive tumor mass. **c** After three fractions of HDR-BT, the tumor appeared almost completely regressed, with only small residual ulcerations and irregularities. **d** Complete endoscopic remission 8 weeks after treatment. The *arrows* indicate minimal erythema and a residual scar

Response assessment (RA) was scheduled 8 weeks after treatment and included MRI of the pelvis and endoscopy. Four of six patients (67%) demonstrated cCR or near cCR at the time of first RA 8 weeks after HDR-BT. One patient presented with a residual but clearly regressive tumor (partial response), while another patient showed residual tumor mass (stable disease) and was subsequently diagnosed with hepatic metastases. Serial endoscopic images of patient 2 are depicted in Fig. 2.

**Fig. 3:**
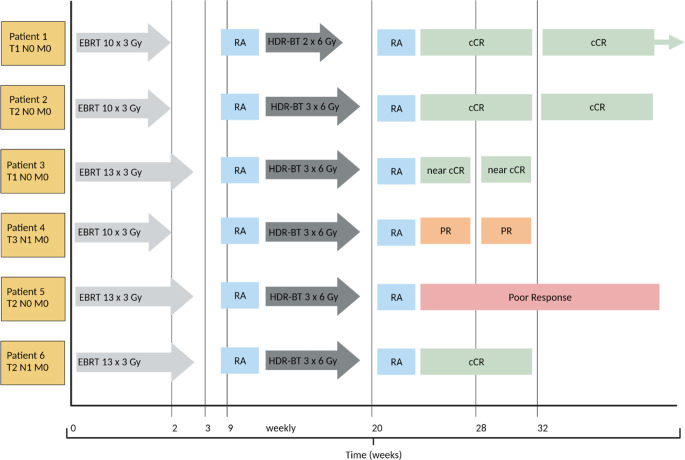
Timeline of the treatment schedule and sequential response assessment (RA) for each individual patient. Patient 1 showed cCR (clinical complete response) after EBRT (external beam radiation therapy). After two of three intended HDR-BT (high-dose rate brachytherapy) fractions, endoscopy confirmed cCR, and the patient refused further treatment. Sixteen months after completion of therapy, endoscopic controls showed sustained cCR. Serial endoscopic images of patient 2 are depicted in Fig. 2. Eight weeks after HDR-BT therapy, the patient presented with a residual scar only, indicating cCR. Three months after completion of therapy, cCR was confirmed. Patient 3 had a small residual ulcer on an initial endoscopic control 8 weeks after completion of HDR-BT, consistent with near cCR. After 8 weeks, a reassessment was performed by endoscopy. The findings were again consistent with near cCR. Extensive endoscopic biopsies showed a low-grade epithelial dysplasia, unspecific chronic inflammation, and significant fibrosis. No tumor cells were detectable. The patient refused any further follow-up. Patient 4 showed partial response (PR) at the first endoscopic control after HDR-BT. After reassessment, following a multidisciplinary decision-making process and due to the general health status, a watch-and-wait/best supportive care (BSC) approach is followed. Endoscopic controls are scheduled. No tumor-related symptoms can be reported. Patient 5 was most likely to have residual scars and posttherapeutic alterations on restaging MRI (magnetic resonance imaging). However, endoscopy showed only a poor local response with obvious residual tumor mass. The patient was diagnosed with histologically confirmed hepatic metastases. Palliative chemotherapy with capecitabine was initiated. Patient 6 presented with cCR 8 weeks after treatment. MRI showed primarily posttherapeutic alterations. No suspicious lymph nodes could be detected

The median follow-up time for all 6 patients is 42 weeks (range 8 to 60 weeks). Follow-up after cCR included endoscopic controls every 3 months. For near cCR, a shortened interval of 8 weeks was provided. Fig. 3 presents RA and follow-up data for each patient. Sustained cCR without evidence of local regrowth has been achieved in all 4 patients with initial (n)cCR to date. Currently, all patients are alive. Colostomy-free survival is 100%. Limited by the still short follow-up period, however, no detailed long-term toxicity profile or functional outcome can be reported yet.

## Discussion

HDR-BT delivers a highly conformal dose distribution, steep dose gradients, and high doses to a confined area [26]. In the preoperative setting, HDR-BT alone or in combination with EBRT has resulted in superior pCR rates compared to EBRT alone [27, 28]. Besides, HDR-BT is associated with a favorable treatment-related toxicity profile [27, 28]. We here report our first experience on EBRT followed by image-guided, adaptive endorectal HDR-BT in a cohort of six rectal cancer patients who were medically unfit for or refused surgery. In this vulnerable patient cohort with a median age of 84 years and significant comorbidities, combination chemotherapy (TNT) or radical surgery is often prohibited, and risks and benefits of treatment should be well balanced.

Preoperative EBRT dose escalation has been explored to increase pCR rates [12, 29]. Dose–response analyses have revealed that an EQD2 of 92 Gy is required to achieve pCR in approximately 50% of patients with locally advanced rectal cancer. In addition, a minimum dose of 72 Gy is considered to be required for major tumor response (tumor regression grading, TRG1-2) in these patients [11]. Previous studies have shown that organ preservation can be achieved by definitive EBRT combined with HDR-BT, whereas results of EBRT alone are limited in this setting [30]. Consequently, Appelt et al. performed radical high-dose chemoradiation with an HDR-BT boost (60 Gy in 30 fractions plus 5‑Gy HDR-BT boost) in a prospective cohort of 55 patients with distal rectal cancer (cT2‑3, N0-1) followed by watch-and-wait (W&W) for intentional organ preservation. Of 51 eligible patients, 40 patients had cCR. At 1 year, local regrowth rate was 15.5%. The ∑EGD2 α/β10 (EBRT + HDR-BT) was 66.3 Gy. The most common late toxicity was rectal bleeding (grade 3 in 2 patients) [31].

In an earlier report by Corner et al., 52 medically inoperable rectal cancer patients were treated with primary (chemo)radiation of 45 Gy followed by HDR-BT of 12 Gy in two fractions at 10-mm depth, or HDR-BT as monotherapy with 36 Gy in six fractions, applied two to three times weekly. Twenty-four patients showed CR (46%) [22]. The comparatively low rate of CR reported by the authors was associated with a low rate of high-grade toxicity. The results reflect on the relatively low EQD2_α/β = 10_ (60.3 Gy) and D_max_ (approximately 85 Gy) at the mucosal level and are in line with the aforementioned dose–response analyses. Chemotherapy may have further disguised the effect of radiation. Furthermore, 2D planning has an increased risk of PTV miss, which may have compromised the clinical outcome.

In the HERBERT phase I dose-escalation trial, 38 medically inoperable rectal cancer (cT2‑3, cN0-2) patients were treated with 39 Gy in 13 fractions followed by three weekly HDR-BT fractions of 5–8 Gy each (∑EQD2 _α/β10_ = 61–78.3 Gy). Of 33 evaluable patients, 20 patients achieved cCR (cCR rate 60%). Tumor regrowth after initial cCR occurred 6 patients, while tumor progression was observed in 6 of initially 9 patients with partial response (PR). Local progression occurred after a median time of 9.3 months [23]. Notably, the authors reported chronic grade ≥ 3 proctitis in 40% of patients. Dose-limiting toxicity was reached at HDR-BT single doses > 7 Gy, so the maximum tolerated dose was set at 7 Gy [32]. Possible reasons for the relatively high rate of toxicities reported by Rijkmans et al. include the following: (1) using 2D treatment planning after initial 3D CT-based treatment planning instead of CT-based planning prior to every fraction could affect PTV coverage, but also OAR hotspot volumes; (2) the prescription depth of 20 mm resulting in very high mucosal contact doses which confirms a correlation between reference dose depth and toxicity [32, 33].

Garant et al. were able to achieve a comparatively low toxicity rate despite an increased ∑EQD2 of 91.7 Gy in a prospectively analyzed cohort of 94 unselected rectal cancer patients (cT1‑4, cN0/+) medically unfit for surgery. After moderately hypofractionated EBRT with 40 Gy in 16 fractions, image-guided adaptive HDR-BT delivering 30 Gy in three fractions prescribed at 10 mm was performed. All patients completed therapy. The increased cCR rate of 86.2% (81/94 patients) supports a strong dose–response relationship. Two-year local control rate was 71.5%. Metastases occurred in 20.2% of patients during follow-up, consistent with data after standard chemoradiation. Long-term toxicity (≥ CTCAE grade 2) occurred in 18 patients (19.2%) [24]. The use of central tungsten shielding and a double balloon system resulted in an asymmetric dose distribution, shifting the PTV surface beyond the 400% isodose and the contralateral rectal wall into the low-dose spillage [34]. A detailed and comparative description of the different EBRT/HDR-BT concepts reported by the previously discussed literature is given in Table 4.

**Table 4:** Comparative overview of treatment characteristics, technical aspects, response, local failure, and toxicity rates for EBRT/HDR-BT reported in the literature

Corner et al. [22]	Garant et al. [24]	Rijkmans et al. [23]	Frankfurt
EBRT 45 (1.8) Gy +	EBRT 40 (2.5) Gy +	EBRT 39 (3) Gy +	EBRT 39 (3) Gy +
HDR-BT 12 (6) Gy	HDR-BT 30 (10) Gy	HD-BRT 21 (7) Gy	HD-BRT 18 (6) Gy
2D planning	CT-based planning	CT-based/2D planning	CT-based planning
BT at 10 mm depth	BT at 10 mm depth	BT at 20 mm depth	BT at 5/10 mm depth
∑EQD2_α/β_ _=_ _10_ = 60.3 Gy	∑EQD2_α/β_ _=_ _10_ = 91.7 Gy	∑EQD2_α/β_ _=_ _10_ = 72 Gy	∑EQD2_α/β_ _=_ _10_ = 66.3 Gy m∑EQD2_α/β_ _=_ _10_ = 76.5 Gy c∑EQD2_α/β_ _=_ _10_ = 146 Gy
Grade 3 toxicity = 6%	Grade 3 toxicity = 19%	Grade 3 toxicity = 40%	–
cCR = 46% Local failure = 21%	cCR = 86% Local failure = 14%	cCR = 60% Local failure = 30%	cCR = 50% (including near cCR = 67%)

∑EQD2_α/β10_ = EQD2_α/β10_ EBRT + EQD2_α/β10_ HDR-BT at 5 mm or 10 mm m∑EQD2_α/β10_ = EQD2_α/β10_ EBRT + EQD2_α/β10_ mean dose HDR-BT c∑EQD2_α/β10_ = EQD2_α/β10_ EBRT + EQD2_α/β10_ HDR-BT contact dose/surface of rectal mucosa *EBRT* external beam radiation therapy, *HDR-BT* high-dose rate brachytherapy, *cCR* clinical complete response

More recently, Garant et al. have presented an interim analysis of the ongoing MORPHEUS randomized controlled phase III trial (NCT03051464) comparing two dose-escalation strategies to achieve cCR in rectal cancer (T2-3ab, N0, M0) based on their previously reported research. After 45 Gy standard chemoradiation, patients were randomized to either an EBRT boost of 9 Gy or a weekly HDR-BT boost of 30 Gy in three fractions. Total mesorectal resection (TME)-free survival was significantly improved in the HDR-BT boost group (85.1 vs. 40.5%, *p* = 0.001), with a cCR rate of 90 versus 50% and a local regrowth rate of 17 and 30%, respectively [35].

In summary, the abovementioned studies highlight the feasibility and good response rates of radically intended radiation therapy combining EBRT and HDR-BT in elderly and frail rectal cancer patients. All studies confirm a strong dose–response relationship. From experience with contact x‑ray therapy, it can be concluded that the mucosal surface of the rectum can withstand doses far beyond 110 Gy EQD2. Local radiation dose escalation enables high rates of local control, but mucosal dose and treatment volume represent crucial variables contributing to a substantial risk of acute and late toxicity [32]. CT-based planning prior to every BT fraction can reduce the risk of PTV miss while target volumes can be directly adjusted [34]. Considering the frailty and comorbidities of this patient population, most of which are associated with limited life expectancy, radical radiation therapy must strike a balance between response rate and toxicities.

Our approach of 13 × 3 Gy EBRT and three fractions of 6‑Gy image-guided endorectal HDR-BT at 5–10 mm incorporates the clinical expertise of other groups. Response and toxicity rates are comparable to existing data in the so far limited sample size. The dose concept and CT-based planning allow precise adaptation of the target volume after imaging and endoscopic demarcation of the residual tumor, minimizing toxicity without compromising treatment efficacy. With an aging patient population, brachytherapy could become an integral part of the landscape of individualized, tailored NOM of rectal cancer, balancing risk and benefit.

## Conclusion

External beam radiation therapy combined with endorectal high-dose rate brachytherapy was feasible and well tolerated as primary non-operative management in elderly/frail rectal cancer patients, providing a comparatively high rate of local control and tolerable toxicity rates.
